# The Da Vinci European BioBank: A Metabolomics-Driven Infrastructure

**DOI:** 10.3390/jpm5020107

**Published:** 2015-04-22

**Authors:** Dario Carotenuto, Claudio Luchinat, Giordana Marcon, Antonio Rosato, Paola Turano

**Affiliations:** 1 Da Vinci European BioBank, FiorGen Foundation, via Sacconi 6, 50019 Sesto Fiorentino, Florence, Italy; E-Mails: dario.carotenuto@gmail.com (D.C.); g.marcon@davincieuropeanbiobank.org (G.M.); 2 Magnetic Resonance Center (CERM), University of Florence, Via L. Sacconi 6, 50019 Sesto Fiorentino, Italy; E-Mails: luchinat@cerm.unifi.it (C.L.); rosato@cerm.unifi.it (A.R.); 3 Department of Chemistry, University of Florence, Via della Lastruccia 3, 50019 Sesto Fiorentino, Italy

**Keywords:** biobanking, metabolomics, standard operating procedures, nuclear magnetic resonance, database

## Abstract

We present here the organization of the recently-constituted da Vinci European BioBank (daVEB, https://www.davincieuropeanbiobank.org/it). The biobank was created as an infrastructure to support the activities of the Fiorgen Foundation (http://www.fiorgen.net/), a nonprofit organization that promotes research in the field of pharmacogenomics and personalized medicine. The way operating procedures concerning samples and data have been developed at daVEB largely stems from the strong metabolomics connotation of Fiorgen and from the involvement of the scientific collaborators of the foundation in international/European projects aimed to tackle the standardization of pre-analytical procedures and the promotion of data standards in metabolomics.

## 1. Introduction

The creation of a biobank infrastructure linking bio-specimens with clinical data generally originates within a clinical context and/or focuses on specific health or disease problems. These infrastructures can generally be reconducted to one of the following formats: Population-based biobanks, disease-oriented biobanks, case-control biobanks, biospecimen specific biobanks (tissues, cord blood, *etc.*), clinical trial related biobanks.

Here, we present the establishment of a methodology-driven biobank, which aims at (although does not limit itself to) the collection of samples and data for future metabolomics analyses and develops protocols and standards that can assure optimal outputs from the use of this analytical methodology. The present case history concerns the creation of the da Vinci European BioBank (daVEB) [1], a small scale infrastructure run by the non-profit Fiorgen Foundation which promotes collaborative interdisciplinary problems in the field of farmacogenomics and personalized medicine. The centralized IT infrastructure, the laboratory and the first biorepository of daVEB are located at the scientific campus of the University of Florence and hosted by CERM (www.cerm.unifi.it). daVEB is a partner biobank in BBMRI-ERIC (Biobanking and Biomolecular Resources Research Infrastructure-European Research Infrastructure Consortium; www.bbmri-eric.eu) and its Italian hub BBMRI.it. In 2011, daVEB obtained quality certification according to UNI EN ISO 9001:2008 for “Collection, storage and distribution of biological samples and the associated data for scientific research” (CSQ Certificate N. 9122.FFFI; IQNet Certificate N. IT-79101); the certification has been renewed in 2014. Procedures and standards adopted at daVEB originate from the strong links with scientists of the University of Florence and their involvement in several EC projects in related fields.

In this article, after a brief presentation of the key aspects of metabolomics in the frame of biobanks, we will review the actions undertaken at daVEB to develop validated procedures for the collection, handling and storage of samples, to provide adequate data sets for the interpretation of metabolomics data through statistical approaches and, last but not least, to facilitate the distribution of the information deriving from metabolomics analyses within the biomedical research community.

## 2. Metabolomics

Metabolomics is the study of the complete pool of small molecules forming the metabolome of a biological sample (biofluid, cell, tissue, or organism). The human metabolome composition accounts for several thousands of small molecules (<2000 Da) produced by the genome of the host organism and by the genomes of its microflora or deriving from the interaction with the environment [2]. The concentrations of the different small molecules, generically called metabolites, span a very ample range, from <1 nM to 1 μM.

The two main analytical methodologies used in metabolomics are mass spectrometry (MS) and nuclear magnetic resonance (NMR). MS-based technologies, which are generally coupled to chromatographic separations, identify the metabolome components based on their charge-to-mass ratio, have high sensitivity and therefore provide a comprehensive overview of the metabolome. As a drawback, they suffer for reproducibility problems especially in quantitative analyses and require specific approaches for chemically different classes of compounds. In NMR-based metabolomics, only the signals of all metabolites present in a given sample above a certain concentration threshold are observable, but they are all simultaneously measured in a single monodimensional spectrum. The information about the chemical identity of many of the observed molecules can be retrieved by matching the spectral patterns to those in available databases. At variance with MS, NMR demands little sample preparation and is rapid and quantitative, but has lower sensitivity [3].

CERM, which hosts the daVEB infrastructure, is the Magnetic Resonance Center of the University of Florence and has a long-standing tradition in NMR of biological systems. Since 1994 CERM has been funded by EC to provide transnational European access to its NMR resources; since 2007 it is one of the core labs of the European Research Infrastructure for Integrated Structural Biology (INSTRUCT; www.instruct-fp7.eu) that has been defined by the European Strategy Forum for Research Infrastructure (ESFRI). During the last decade and in collaboration with the Fiorgen Foundation, CERM has established a metabolomics research unit/group and hosts NMR spectrometers that are fully dedicated to metabolomics analyses. Collaborative projects have led to the metabolomics characterization of several pathological states (celiac disease, heart failure, and different types of cancer and other diseases) [4,5,6,7,8,9,10,11,12,13,14,15,16,17,18]. Very importantly the existence of an individual fingerprint in the urine metabolome of a group of subjects has been identified, which is stable over the time course of years [19,20]. The identification of specific disease signatures in the metabolomics profiles and the ability to detect drifts in the individual metabo-phenotype are expected to have potential applications for early diagnosis [21].

### Metabolomics for Biobanks and Biobanks for Metabolomics

There is an obvious link between metabolomics and biobanking activities: Metabolomics profiling and biomarker discovery rely on the comparison of data acquired on large ensembles of samples from different groups of donors. The subsequent analysis allows researchers to assess group-wise differences with statistical approaches that are mainly adapted from earlier emerged omics technologies. Biobanks are obvious providers of samples for large-scale studies and, as such, are a much-needed resource in metabolomics, adding capacity and creating value for the research community.

The biomedical significance of metabolic analyses requires that the metabolome of the sample to be studied represents as closest as possible the original “*in vivo*” metabolome. However, during the entire pre-analytical phase (which covers sample collection, transport, handling, and storage) the concentrations of several metabolites can change due to residual enzymatic activities in the samples and chemical reactions, e.g., induced by exposure to air and/or light. The collaboration between CERM and daVEB has led to the development of metabolomics as an analytical tool to evaluate the effect of different pre-analytical procedures on the sample metabolome [22,23,24]. Several of these activities were carried out in the context of the FP7 project SPIDIA (http://www.spidia.eu/) and have led to development of evidence-based standard operating procedures, now implemented at daVEB, which cover the pre-analytical steps for urine, serum and plasma samples [22,24]. In particular, best practices for urine require that the collected sample is kept refrigerated (4 °C) for a maximum of 2 h and freezing is avoided prior to mild centrifugation and/or filtration to prevent cell disruption upon ice crystal formation [22,24]. Analogously, it has been found that a tight control of temperatures and time during the pre-processing of blood and before freezing of the derived serum and plasma samples is important to preserve the enzymatic degradation and the oxidation of several of the most abundant metabolites [24]. Some critical steps of tissue preanalytics have been also addressed [20]. For these samples pre- and post-excision ischemia times were demonstrated to be important sources of variability in metabolic profiles. Preservation of sample properties throughout the long-term storage at daVEB is under study; the definition of the maximum life time of a sample in terms of the stability of its small molecule profile has obvious implication for the outcome and reproducibility of metabolomics studies based on samples stored in biobanks and impacts on the budget of biobanks because of the costs associated to sample storage.

For the close links between metabolomics and biobanks, researchers at CERM have promoted the foundation of consortium called EXCEMET (EXpert CEnter for METabolomics; www.excemet.org) [25], aimed at providing metabolomics services to the previously cited BBMRI-ERIC. As of today, eight parties are members of the consortium: Consorzio Interuniversitario Risonanze Magnetiche di Metallo Proteine—CIRMMP (Florence, Italy), Medical University of Graz (Graz, Austria), Metanomics Health GmbH (Berlin, Germany), University of Gothenburg (Gothenburg, Sweden), University of Birmingham (Birmingham, UK), Helmholtz Zentrum München Deutsches Forschungszentrum für Gesundheit und Umwelt—GmbH (München, Germany), Steno Diabetes Center (Gentofte, Denmark), EMBL-EBI (Hinxton, UK).

## 3. daVEB Collections

**Figure 1:**
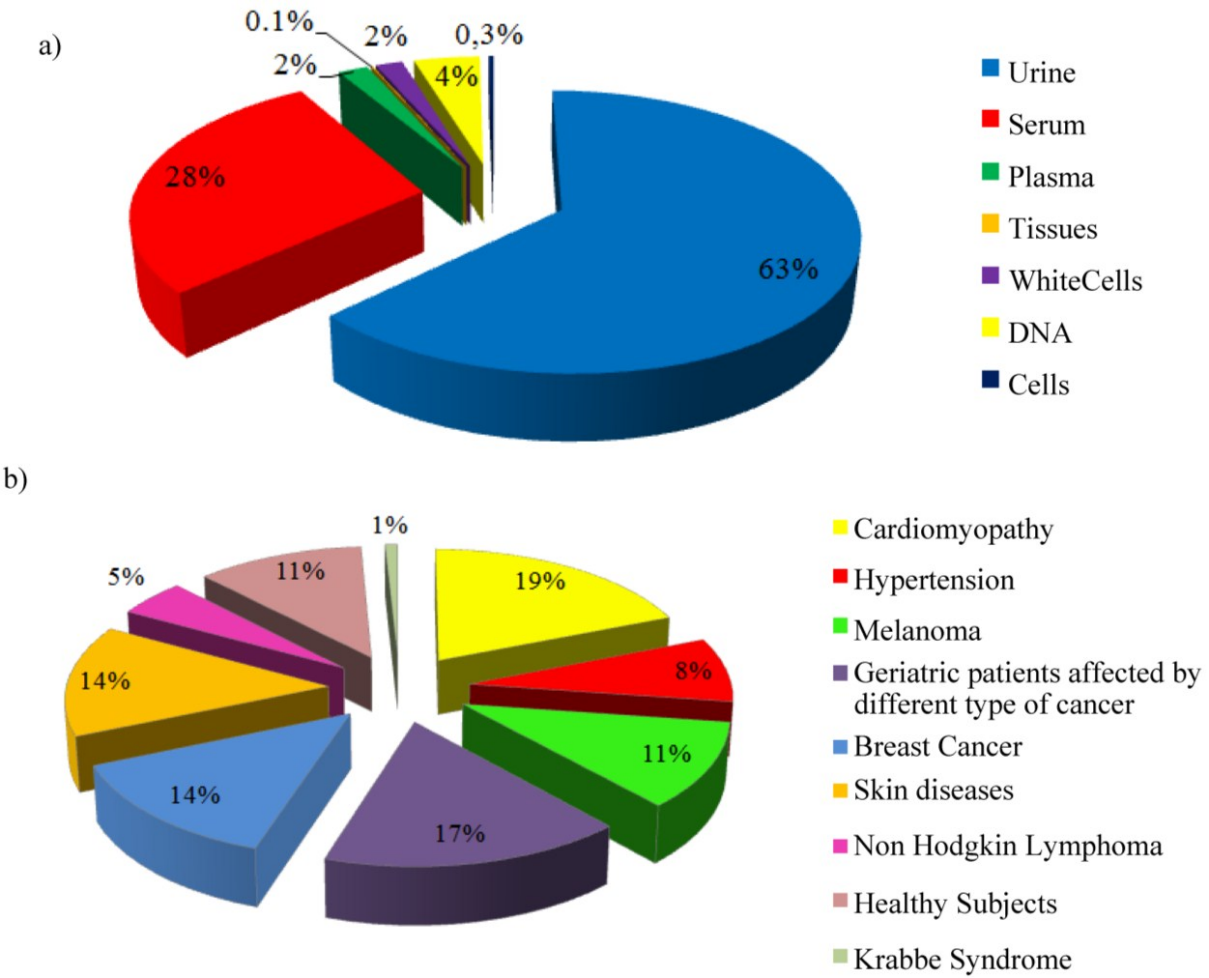
Percentage distribution of da Vinci European BioBank (daVEB) collections by (**a**) samples type and (**b**) pathology (healthy subjects included).

The biomaterial available at daVEB includes different types of samples, as summarized in Figure 1a, with a strong focus on serum and urine, as the most common biofluids employed in metabolomics studies. The samples arise from patients with different pathologies (Figure 1b), but an ample collection comes from healthy controls, which are essential to develop models of metabolomic fingerprint of diseases. Donors defined as healthy subjects are individuals that meet the criteria for voluntary blood donation. A more detailed description of the daVEB sample collections is provided as Supplementary Material (see Table A1).

The availability of gender and age-matched controls is an added value for the definition of the diseases signature in metabolomics profiles. The present capacity of the biobank is of about 265,000 vials (0.5, 1 and 2 mL in volume) to be stored at −80 °C and 195,000 vials (0.5, 1 and 2 mL in volume) to be stored in the vapor of liquid nitrogen, at temperatures below −170 °C. Considering the sample turnover due to sample distribution for research purposes, on average daVEB hosts about 10,000 samples.

The biobank catalogue can be browsed via the web: https://www.davincieuropeanbiobank.org/en/research/sample-search. Samples and the associated data, which are project specific, are available for research purposes to external users, according to the published accession criteria [1].

A minimum data set is required for a sample to be accepted in daVEB, which includes the following info: Phenotypic descriptions (donor height, weight, BMI), demographic information (gender, age, ethnicity), individual information (pregnancy, breast-feeding, post-menopausal woman, fasting, smoking habits, alcohol consumption, disease or healthy status, infection state).

Information concerning the sample history is associated to each aliquot, in particular the processing SOP followed at collection, the temperature story (freeze chain) including transport and storage temperatures, and the freeze time, from transport date and duration, entry date in −80 °C mechanical freezers or tank for storage in the vapor phase of liquid nitrogen, to the registration and exit date.

## 4. High Standards in the Data Management System

The technology-driven nature of the da Vinci European BioBank raises the need for a very specific design of its IT infrastructure. In particular, the present design takes into account the following characteristics of the operations of daVEB: (a) the samples stored originate from different collections, which do not share a focus on a common disease nor a common sample type and are collected in different, multiple centers; (b) each collection is associated to a different organization and a different ensemble of clinical data; (c) besides clinical data, samples are associated to other biomedical information, which, in addition to metabolomics data (present for the large majority of samples), can include genetic data, various kinds of images, *etc.* These three characteristics made it necessary for the daVEB biobank to implement a flexible infrastructure permitting the management of different data types for different collections, both for clinical and for other biomedical information. Furthermore, the IT infrastructure allows each collection to be extended at a later moment to include new samples (e.g., for follow-up) or new data types to be associated to the current samples (e.g., the results of analyses not initially available). A further need arising from the above is to implement an approach to search our database that allows users to compare collections with different content types and, sometimes, formats.

A paramount concern when dealing with biobanks is the secure access to the data and the confidentiality of personal data. The daVEB IT infrastructure has addressed this problem by consistently Open SSL/TSL standard protocols for data exchange and network communications. Furthermore, data are separately stored onto two different servers. All personal information is kept on a server that is not accessible from the public network, but only through a private network and with different credentials than the first server. All other data, which do not raise privacy issues, reside on a separate server reachable via the public network.

For the practical implementation of the daVEB IT infrastructure, we decided to exploit as much as possible open standards and open source software. This should also facilitate the interaction, and eventually the exchange of information, with other biobanks at the European and global level. Our data models are thus available in the form of schemes stored as Open Document Format (ISO Standard) data sheets; as an example, this includes the specific formats and definitions adopted for the information concerning sample history. Regarding data vocabularies and semantics, we designed procedures that allow the daVEB staff to define relationships between different relevant ontologies. We centrally rely on the Open Biological and Biomedical Ontologies made available by the OBO foundry (http://www.obofoundry.org/) [26]. However, other ontologies are also acceptable, if they can be accessed as RESTful services [27]. This makes it possible, for example, to use different catalogues of standard disease codes. Links between the corresponding entries of different catalogues can be established in a semi-automated fashion and then subject to manual curation and editing. To define the typical information content associated to a collection and its samples, we took into the core variables defined within the DataSHaPER approach [28]. This should facilitate the harmonization of our data with other studies, also not involving biobanks. On top of this common background, the data schema can be tuned for each sample collection, as detailed in the next paragraph. Finally, we deployed the database management and operating system components using PostgreSQL DBMS and CentOS Linux, respectively. Specific backup and update procedures have also been defined. daVEB is equipped with a fiber channel connecting to a HP MSL6030 Tape Library with two LT04 drives. We implemented a backup schedule that balances lengthier full backups and faster incremental backup strategies, with weekly full backups and daily incremental backups. Moreover, every two months we move to a separate location a full backup of all daVEB data.

**Figure 2:**
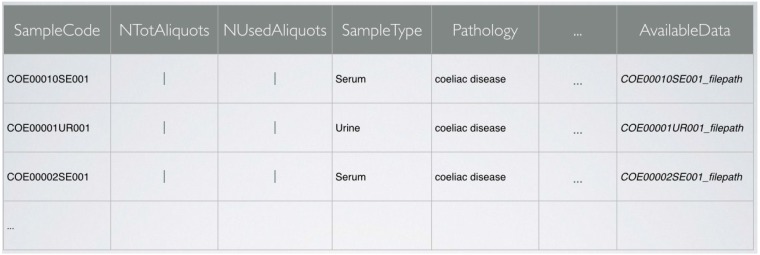
Scheme of the data table for a collection of samples from coeliac patients. The SampleCode field uniquely identifies each sample of the collection.

More in detail, each collection corresponds to a specific data scheme. An example is shown for a collection of samples from coeliac patients as a table in Figure 2. In the example, to each sample of the collection the system associates SampleCode, Pathology, the number of aliquots available and the number of aliquots already used (e.g., for metabolomics experiments). The field AvailableData contains a link to the collected metabolomics data. Additional such fields can be added to the table, also after its creation, to point to other data types, such as genetic profiling. Additional tables are used in a similar fashion to store the data of donors as well as clinical data.

**Figure 3:**
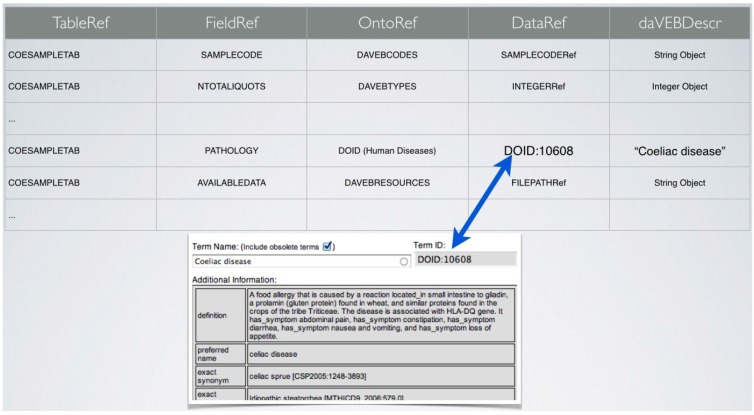
Example of first-level daVEB metadata table.

The meaning of the fields in a table such as that of Figure 2 is defined in higher-level metadata table, as shown in Figure 3. Such table contains the definition of the metadata associated to each collection of the daVEB biobank. This is done by defining for each field of the data table of the collection the name of the field, its reference to a specific ontology, and the type of data (numeric, string, link, *etc.*) that can be associated to each field (see Figure 3). Each record of the metadata table corresponds to a specific field (defined by the combination of TableRef and FieldRef fields). The OntoRef field indicates the ontology used for that particular field of the data table, which can be an internally defined ontology or a public reference ontology. The reference used for the field is indicated by the DataRef field, whereas the allowed data type is defined by the daVEBDescr field. Each data type, as mentioned, can be a single instance field, e.g., “Coeliac Disease”, or a type set. If the ontology used is an external one, then the DataRef field can point directly to external RESTful ontology, as shown in the example. This allows the unequivocal definition of the term “Coeliac Disease”. However, it is possible to use multiple ontologies, if necessary, by providing multiple records for each term, with pointers to the appropriate external vocabularies.

**Figure 4:**
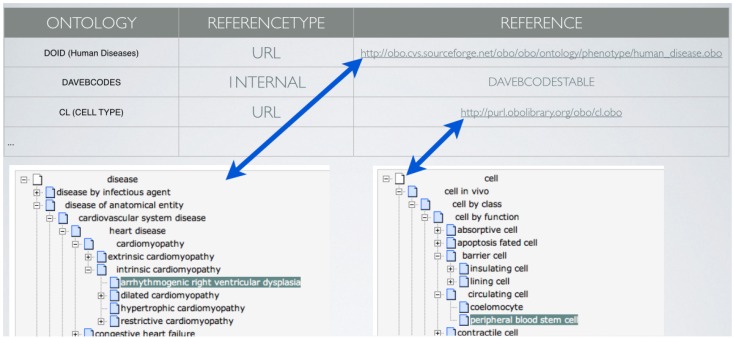
The daVEB Ontologies table.

At the top of the metadata tables of the daVEB stack there is the Ontologies table (Figure 4), where every single ontology and standard data set is referenced through an appropriate HTTP path to allow remote procedure calls exploiting RESTful services.

Let us now consider the case of a user who wants to compare the data contained in two different collections. Such projects will have different data tables and descriptors. In a more traditional setup than that of the daVEB biobank, the corresponding data would most likely be collected into different databases adopting different data schemes. This however would make it difficult and inefficient to merge queries over the two collections of interest in order to discover, for example, similarities/differentiations among the life habits of donors. The daVEB approach allows us to combine queries for each single project into a single query using the standard SQL language for queries. Such queries exploit the relations among metadata definitions contained in the local tables or accessible via public RESTful services (see Figure 4).

Therefore, we can easily define a query relating data among two different projects, e.g., the COE and MET projects:

*find all*

(A) diet habit elements for healthy subjects in MET project

*that are not in*

(B) diet habit elements for pathologic subjects in COE project

We can translate such a question into a nested SQL query using the data contained in metadata tables to capture common definitions for diet habit in the MET and COE projects, and use such information to determine the result set, defined as the difference A-B. The entire data extraction procedure is automated and entirely SQL driven. 

As far as the development of standards for the inclusion of metabolomics data in the biobank database, the involvement of some of us in the FP7 project COSMOS has been particularly valuable. COSMOS is a collaborative project funded by the European Commission within the 7th Framework programme (http://www.cosmos-fp7.eu/). The specific aim of COSMOS is to develop open standards for the exchange of metabolomics data and the associated metadata, together with a variety of tools to support capturing and management of such information. These tools interface to the software used for data acquisition, to commonly used programs for the analysis of metabolomics data, and to relevant databases, such as Metabolights (http://www.ebi.ac.uk/metabolights/) [29]. COSMOS has been developing its standards in collaboration with the major instrumentation vendors as well as publishers in the field, and relevant international organizations. As mentioned, a key specificity of the daVEB biobank is the availability of NMR-based metabolomics information for the large majority of its samples, especially urine and blood. The interaction with the COSMOS project is thus instrumental to make such information as widely reusable as possible. In this respect, the development of nmrML (http://nmrml.org) as on open mark-up language specifically designed for NMR data resulting from metabolomics applications has been a crucial achievement. Notably, it is not needed to store the experimental data in the nmrML format, as they can be directly imported at a later stage. On the other hand, the ontologies used within the daVEB data warehouse are compatible with the ISA software suite that is central to metadata management in COSMOS. COSMOS has been developing a controlled vocabulary for NMR experiments, called nmrCV, adopting the OWL (Web Ontology Language, developed by the W3C consortium) syntax. The OBO ontology used within the IT infrastructure of daVEB can be readily converted to the OWL format.

Beyond the mere distribution of the metabolomics profiles associated with the various samples, the daVEB biobank provides support for the reuse of the derived biomedical and clinical information. For example, COSMOS provides a computational infrastructure to identify and quantitate specific metabolites within the entire profile. This information can be derived from the metabolomics data in the daVEB biobank through a variety of analytic approaches and its significance is defined on a statistical basis [30]. However, this requires the involvement of an expert skilled in metabolomics to perform the analysis. In the future it may become feasible for daVEB users to directly access the derived information, through the COSMOS framework, thus avoiding the need for repeating the computational analysis, unless desired/needed.

## 5. Conclusions

The rapid development of high-throughput technologies and computational frameworks enables the examination of biological systems in unprecedented detail, paving the way to significant advances in personalized and precision medicine. By providing tissues, cells, fluids, and raw data, biobanks have become the drivers of the translational research cycle, since they are the custodians of the biospecimens and related anonymized data. A deep knowledge of the requirements inherent to a specific omic analytical approach allows biobanks to foster the development of this discipline. The close collaboration between biobanks and analytical platforms becomes essential to create a new generation of biobanks acting as drivers of the development of new methodologies.

## Appendix

**Table A1:** The different kind of biosamples available for each disease, as absolute numbers.

Disease	Donor number	Sample type	Sample number	Available aliquots
**Cardiomyopathy**	291	Urine	776	2863
		Serum	810	2492
**Hypertension**	126	Serum	126	378
**Melanoma**	156	Urine	153	892
	169	Serum	174	1137
	166	Plasma	170	1132
	154	White Cells	156	454
	4	Tissue	5	12
**Geriatric patients affected by different type of cancer**	257	Urine	514	941
		Serum	257	263
**Breast Cancer**	209	Urine	1520	2150
		Serum	735	412
**Skin diseases**	219	Serum	219	254
	2	Plasma	2	2
	21	Cells	27	27
	3	Tissue	3	3
**Non Hodgkin Lymphoma**	73	DNA	381	402
**Healthy Subjects**	175	Urine	2476	9604
		Serum	149	170
**Krabbe Syndrome**	13	Urine	10	79
		Serum	11	45
